# Structural basis to repurpose boron-based proteasome inhibitors Bortezomib and Ixazomib as β-lactamase inhibitors

**DOI:** 10.1038/s41598-022-09392-6

**Published:** 2022-04-01

**Authors:** Markus Perbandt, Nadine Werner, Andreas Prester, Holger Rohde, Martin Aepfelbacher, Winfried Hinrichs, Christian Betzel

**Affiliations:** 1 Institute for Biochemistry and Molecular Biology, University of Hamburg, Hamburg, Germany; 2 Institute for Medical Microbiology, Virology, and Hygiene, University Medical Center Hamburg-Eppendorf, Hamburg-Eppendorf, Germany; 3 Institute for Biochemistry, University of Greifswald, Greifswald, Germany; 4 Present Address: Department of Integrated Structural Biology, Centre for Integrative Biology, IGBMC (Institute of Genetics and of Molecular and Cellular Biology), Illkirch, France

**Keywords:** Drug discovery, Structural biology, Antimicrobials

## Abstract

β-lactamases are a major cause of rapidly emerging and spreading antibiotic resistance. Currently β-lactamase inhibitors (BLIs) in clinical use act only on Ambler Class A, C and some class D lactamases. The urgent need to identify new BLIs recently lead to FDA approval of boron-based compounds BLIs, e.g. Vaborbactam. The boron-based proteasome inhibitors Bortezomib and Ixazomib are used in cancer therapy as multiple myeloma drugs but they also bind to Ser-/Thr- proteases. In this study we show the crystal structures of the β-lactamase CTX-M-14 with covalently bound Bortezomib and Ixazomib at high resolutions of 1.3 and 1.1 Å, respectively. Ixazomib is well defined in electron density whereas Bortezomib show some disorder which corresponds to weaker inhibition efficiency observed for Ixazomib. Both inhibitors mimic the deacylation transition state of β-lactam hydrolysis, because they replace the deacylating water molecule. We further investigate differences in binding of Bortezomib/Ixazomib to CTX-M-14 and its target proteases as well as known β-lactamase drugs. Our findings can help to use Bortezomib/Ixazomib as lead compounds for development of new BLIs.

## Introduction

β-lactam antibiotics are the most frequently used antibacterial agents and have revolutionized the clinical therapy of bacterial infections. The four main classes of β-lactams include penicillins, cephalosporins, carbapenems and monobactams, all of them based on the four-membered 2-azetidinone ring^1^. β-lactams inhibit penicillin-binding proteins (PBPs) like D-alanyl-D-alanyl-transpeptidases which crosslink the peptide side chains of peptidoglycan strands as crucial step during bacterial cell wall biosynthesis^2^. The β-lactam ring reacts to an acyl-enzyme complex that is stable against cleavage by an acceptor or hydrolysis. In that way the β-lactam moiety mimics that of the D-Ala-D-Ala subunit^3^ and acts a substrate analogue.

The clinical usefulness of β-lactam-based antibiotics has been significantly shortened by the emergence of specific bacterial enzymes referred to as β-lactamases (BL), able to hydrolyze and functionally inactivate β-lactam antibiotics. Today, β-lactamases are the most common resistance mechanism in Gram-negative bacteria, and over eight decades of β-lactam usage bacteria have evolved an extraordinary number of variants (> 2000 reported to date)^4,5^. Of worldwide concern is the recent spread of β-lactamases exhibiting hydrolytic activity even against carbapenems, being essential last resort antibiotics used to treat life-threatening infections^6^. BLs are usually categorized according to their sequence into four Ambler classes (A-D)^7,8^. Classes A, C and D are serine-reactive β-lactamases (SBLs), whereas class B enzymes (metallo-β-lactamase, MBLs) require one or two Zn^2+^ ions for β-lactam hydrolysis and likely have a different evolutionary origin^9^. Among the class A, the extended-spectrum SBLs (ESBLs) which are able to hydrolyze cephalosporins and monobactams represent a public health concern^10–12^. The highest number of variants for ESBLs belong to the CTX-M family. The exponential growth of CTX-Ms worldwide has been referred as the “CTX-M pandemic”^10,13^. The variants CTX-M-15 and CTX-M-14 are by far the most important isotypes spreading in humans and animals^14,15^.

BLs are inhibited by β-lactam inhibitors (BLIs), e.g. clavulanic acid, tazobactam, and sulbactam^16–18^. These BLIs, although itself being β-lactams, form a stable acyl-enzyme complex, thereby protecting BLs from hydrolysis and enable for their clinical use. Of key clinical significance, however, emerging BLs (e.g. ESBL or carbapenemases) are not or not sufficiently inhibited by those first generation BLIs. This prompted great efforts to develop new families of BLIs that are also effective against many problematic lactamases. Avibactam, a diazabicyclooctane derivative (DBO), was the first representative of these new non-β-lactam BLIs and forms a relatively hydrolytically stable acyl-enzyme type complex^19^. In contrast to earlier generation inhibitors avibactam binds reversibly allowing re-cyclization and inhibition of additional β-lactamase molecules. A closely related compound, Relebactam, was approved by the FDA in 2019. Similar to Avibactam, Relebactam has a diazabicyclooctane derivate core, but nourished by a piperidine ring^20,21^. Both inhibit Ambler class A and C and some class D β-lactamases, but not MBLs.

A second novel approach for BLI development is represented by boronic-acid based compounds. The ability of boric acid to react as Lewis acid to adopt tetrahedral geometry enables it to effectively mimic tetrahedral transition state analogues during the hydrolysis of acid-amide bonds of peptides or β-lactams^22^. Penicillins and cephalosporins had been modified to displace the azetidinone ring by a boronic acid to create chiral glycylboronic acids^23^. Since then, variations of the carboxylate recognition site of the first glycylboronic acids yielded many inhibitory compounds^24^. Recently the development of cyclic boronates as broad-spectrum inhibitors of β-lactamases has resulted in two novel BLIs Vaborbactam^25^ and Taniborbactam, which have been approved or are in a late stage of development. More than 300 boron-containing compounds in complex with their target proteins have been deposited in the PDB^26^. Serine proteases represent by far the most common class of targets. The transformation of a protease substrate into a boron-based inhibitor involves replacement of the scissile amide bond by the boronic acid moiety^27^. In general, boron is one of the most useful elements in synthetic organic chemistry and the approval of other boron-based medicines, such as Bortezomib and Ixazomib used in cancer therapy as multiple myeloma drugs underscore the potential of boron in drug discovery^28–30^. Like Vaborbactam/Taniborbactam the bioactive form of Bortezomib/Ixazomib is the free boronic acid, which binds to the active site serine as a Lewis acid. However, Bortezomib/Ixazomib have been shown to bind to threonine proteases^31^ and serine proteases^32–34^ whereas Vaborbactam/Taniborbactam binds to the active site of β-lactamases^25,35,36^.

For many years it is well known that β-lactams can be utilized as serine protease inhibitors^37^. The active sites of serine proteases and serine β-lactamases share structural key features mandatory for the catalytic mechanism. This encouraged us in our strategy to identify and characterize known protease inhibitors and approved drugs as possible dual-mode inhibitors. We have utilized class A enzyme CTX-M-14 from *Klebsiella pneumoniae* as a model system, because it is the most widely distributed extended-spectrum β-lactamase (ESBL) globally^14,15^. Here, we describe inhibition assays and crystal structure analyses of CTX-M-14 in complex with protease inhibitors Bortezomib and Ixazomib at near-atomic resolution of 1.3 Å and 1.1 Å, respectively. Our results provide deeper insight to the chemical nature of the inhibition and prove Bortezomib and Ixazomib as lead structures to develop novel β-lactamase-inhibitors.

## Results and discussion

### High resolution structures of Bortezomib and Ixazomib inhibitors of the serine β-lactamase CTX-M-14

We determined the crystal structures of CTX-M-14 in complex with Bortezomib and Ixazomib at a near-atomic resolution of 1.3 Å and 1.1 Å, respectively, to explore the structural basis for the inhibition. As reference we determined the substrate-free enzyme at atomic resolution (1.0 Å) to identify possible changes upon inhibitor binding. The crystallographic asymmetric unit contains one monomer and the active site of all molecules is solvent accessible and not blocked by crystal packing. The solvent channels are large enough to allow ligand diffusion from the crystal surface to the active sites and therefore making this crystal form perfectly suitable for successful soaking experiments. The structure of the native enzyme can be super-positioned with the Bortezomib and Ixazomib complex with root-mean-square deviations (RMSD) of 0.11 and 0.14 Å, respectively, using all atoms of the polypeptide and without remarkable positional deviations of any residues. No induced-fit mechanism upon inhibitor binding takes place and all active site residues remain in the position as in the substrate-free enzyme. The overall data and model quality is extraordinarily high and summarized in Table S1. Both inhibitors were introduced by soaking protocols with a final end-concentration of 20 mM. Based on the high resolution and the quality of the data, we were able to refine and to compare the occupancy of Bortezomib and Ixazomib bound to the active site. Both inhibitors are observed with full occupancy, but the N-terminal end of Bortezomib is disordered due to conformational alternatives. This is in line with the lower inhibition rate of CTX-M-14 by Bortezomib compared to Ixazomib as discussed below. However, together with the so far reported co-crystal structures, these crystallographic studies reveal structural insights for the dual inhibition of both proteases and β-lactamases.

### Bortezomib and Ixazomib mimic the deacylation transition state and reveal reasonable inhibition activity

**Figure 1:**
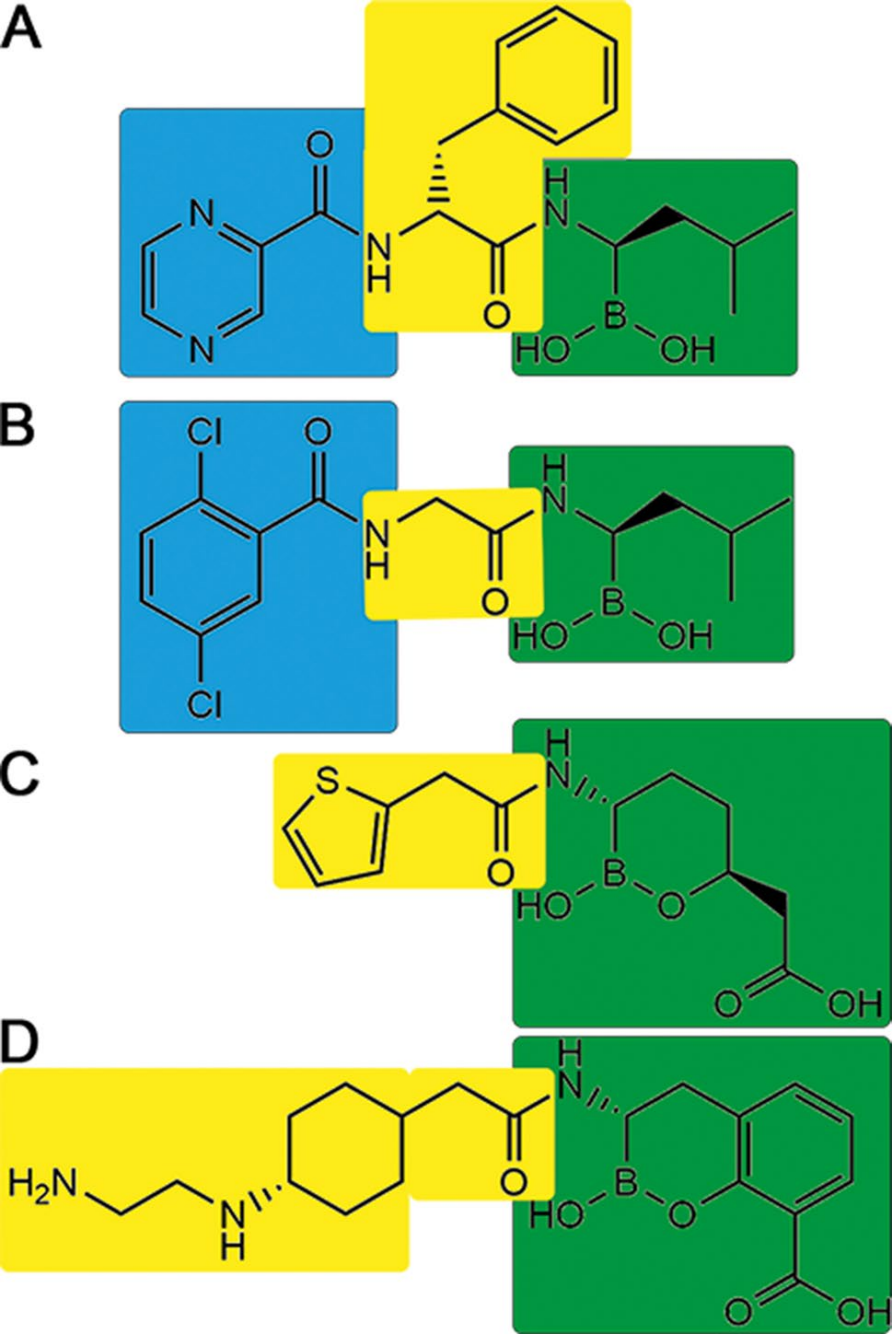
Structural diagrams of the N-protected tripeptide Bortezomib (**A**) and Ixazomib (**B**), and of the cyclic α-acylamino-boronate based β-lactamase inhibitors Vaborbactam (**C**) and Taniborbactam (**D**). All four are peptidomimetics imitating a di- or tripeptide core with a central amide group (highlighted in yellow) flanked by a boronic-acid derivative (in green). The pyrazine (Bortezomib) and dichlorBz (Ixazomib) moieties (highlighted in blue) are missing counterparts in Vaborbactam/Taniborbactam.

Both peptidomimetic inhibitors share a high degree of similarity as N-protected dipeptides and mimic a peptide substrate based on a boronate with a tripeptide-like structure (Fig. 1). Ixazomib is a derivative and a successor of the Bortezomib as proteasome inhibitor. Bortezomib can be written as Pyz-Phe-boroLeu, which stands for pyrazinoic acid, phenylalanine and leucine with a boronic acid instead of a carboxylic acid. Ixazomib is abbreviated as dichlorBz-Gly-boroLeu, which stands for 2,5-dichlorbenzoic acid, glycine and leucine with a boronic acid instead of a carboxylic acid. Both compounds are structurally similar to already known glycylboronic acids^23^. These boronic acids are especially useful for probing recognition in that they are reversible inhibitors, forming fast-on/fast-off Lewis acid adducts with serine β-lactamases.

**Figure 2:**
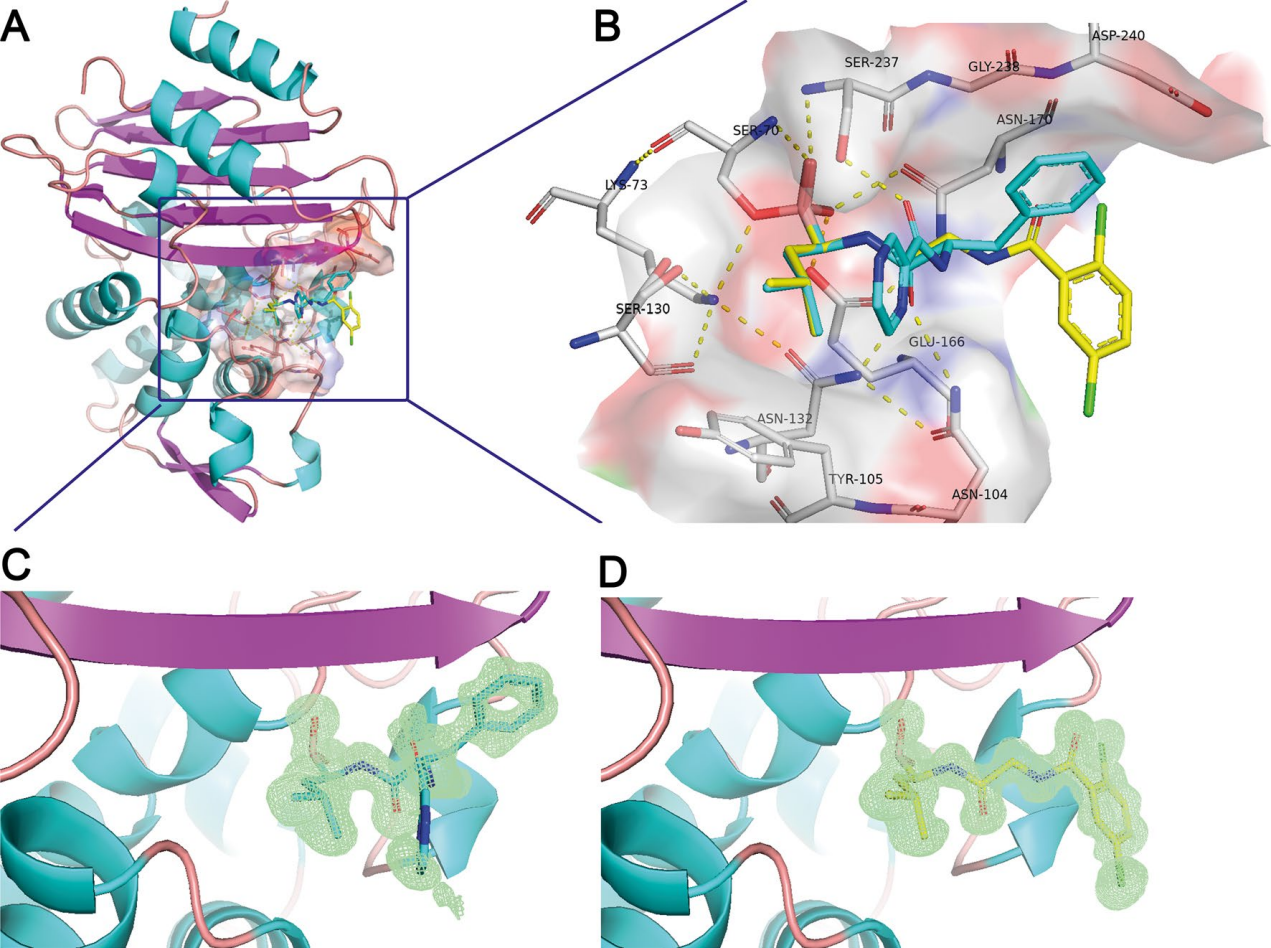
Ribbon plot of the overall structure of CTX-M-14. The active site region is highlighted by a surface representation and the active site residues and covalently bound Bortezomib/Ixazomib is shown as sticks (**A**). The close-up of the active site region (**B**) reveals the hydrogen bonding network among the active site residues. The covalently bound inhibitors are super positioned and shown in sticks with C-atoms colored in cyan and yellow for Bortezomib and Ixazomib, respectively. The corresponding *F*_o_—*F*_c_ polder omit maps of the inhibitors in complex with the refined models of CTX-M-14 are shown in light green and contoured at 3σ (**C** and** D**).

Bortezomib and Ixazomib bind to the active site of CTX-M-14 (Fig. 2a, b) to the catalytic side chain of Ser70 forming an ester with the boroLeu warhead. The borate ester mimics the tetrahedral acylation intermediate in the oxyanion hole. Furthermore, both inhibitor structures are consistent with a deacylation transition state analogue of β-lactam hydrolysis^38^, because they replace the so-called catalytic water molecule (see below).This is most interesting, because the vast majority of known boronic acid inhibitor complexes resemble only the acylation transition state. So far only chiral glycylboronic acids^23,24^ and 3-nitrophenyl boronic acid^39^ are identified as deacylation transition state inhibitors.

In the acyl-enzyme the oxyanion hole stabilizes the tetrahedral transition state by hydrogen bonds of the substrate with carbonyl oxygen and amide groups of the enzyme^40^. One hydroxyl group (O1) of the boronate is bound to the oxyanion hole, accepting two hydrogen bonds of the main chain nitrogen atoms of Ser70 (2.8 Å) and Ser237 (2.9 Å). The other hydroxyl group (O2) of the boronate replaces the catalytic water molecule and contributes to the extended hydrogen network. This hydroxyl is hydrogen bond donor for the carbonyl of the Asn170 side chain, but acceptor for the carboxylic acid side chain of Glu166. The hydrogen-bonding-network is further stabilized by a hydrogen bond donated by the carboxamide nitrogen of Asn170 to the carboxylic-acid group of Glu166 (2.9 Å). The hydrogen bonding pattern is in good agreement with very recently reported ultra-high-resolution X-ray and neutron diffraction crystal structures of other SBLs^41,42^.

The amide moiety of boroLeu of Bortezomib and Ixazomib form important interactions, donating a hydrogen bond to the Ser237 backbone (3.2 Å) and accepting hydrogen bonds from the side chains of both Asn132 (2.9 Å) and Asn104 (3.0 Å). These residues are largely conserved in β-lactamases of class A and C. Furthermore, Asn132 side chain is donating an additional hydrogen bond to the side chain of Asn104 (2.9 Å) and strengthens the hydrogen-bonding network. The hydrophobic leucine side chain in Bortezomib and Ixazomib reveals van der Waals contacts to Tyr105 and Ser130 and is thus shielded from the solvent from one side. The other side is in contact to the Pyz ring of Bortezomib, but solvent exposed with Ixazomib. We see a different conformation of Bortezomib beginning at the central Cα atom of the inhibitors. The Gly residue of Ixazomib corresponds to a voluminous Phe residue and shows therefore minor steric hindrance. Obviously, the Gly residue of Ixazomib fits better into the S2 pocket than the corresponding Phe of Bortezomib. As a consequence, the terminal residues of the inhibitors dichlorBz and the corresponding Pyz are accommodated in very different positions (Fig. 2b). The dichlorBz moiety of Ixazomib occupies a solvent accessible pocket in CTX-M-14, different to the phenylalanine moiety and the terminal Pyz ring of Bortezomib (Fig. 1b). Ixazomib is completely well ordered and well defined in the electron density maps (Fig. 2d). This is not the case for Bortezomib. The Pyz-Phe moiety is less well defined in the electron density map (Fig. 2c). Obviously, the Pyz-Phe moiety is not stabilized by any specific interactions and can adopt alternative but quite similar conformations. The Phe reveals non-polar interactions with Gly238 and Asp240. The Pyz ring occupies a similar space as the acyl group of several β-lactams and is highly solvent exposed. We assume that these parts only insignificantly contribute to the bonding capacity of the compound to the enzyme. This opens the chance to modify a drug with enhanced specificity.

**Figure 3:**
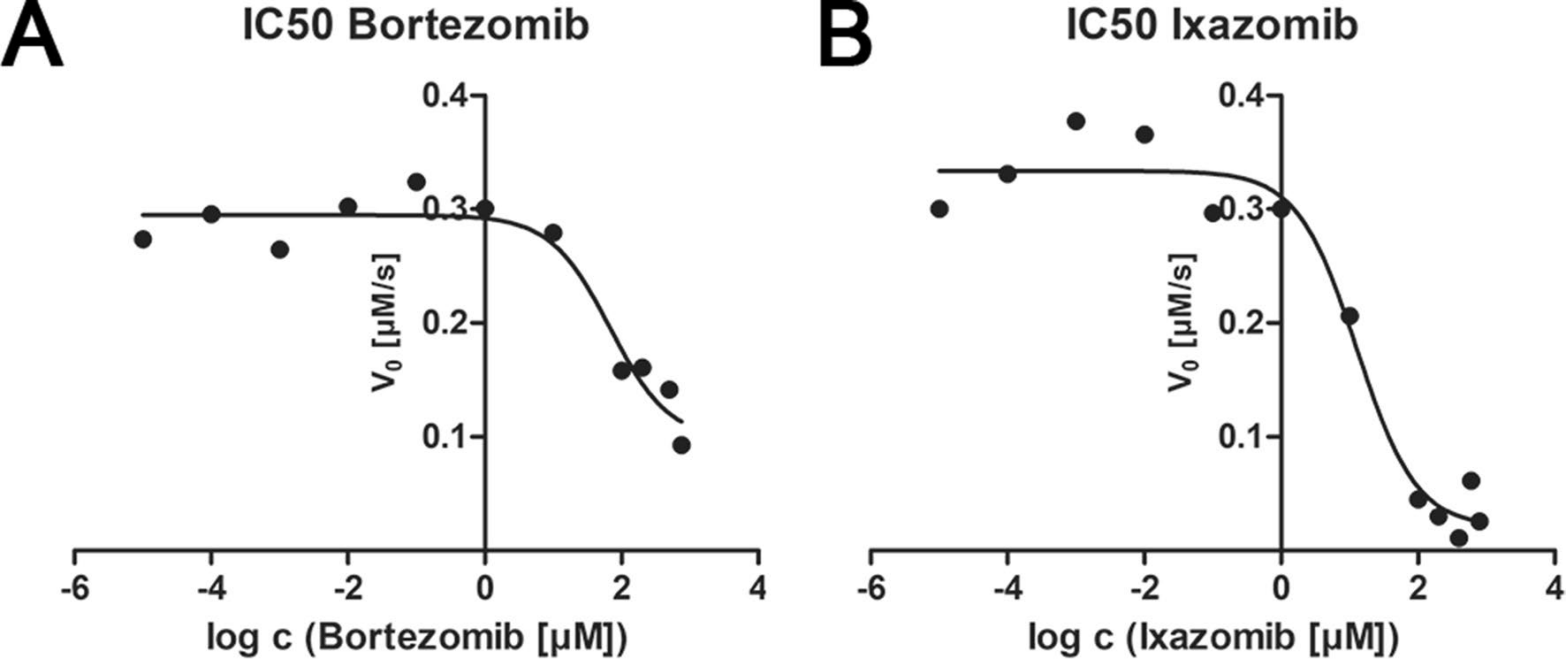
Inhibition assays showing the effect of Bortezomib (**A**) and Ixazomib (**B**) on β‑lactam hydrolysis by CTX-M-14 β‑lactamase. Initial rates of Cefotaxime hydrolysis are plotted against the log concentration of Bortezomib and Ixazomib, showing the decrease of hydrolysis rates at higher compound concentrations, resulting in calculated IC_50_ values of 67 ± 1.4 µM for Bortezomib and 13 ± 1.2 µM for Ixazomib.

The structural data are in line with the biochemical inhibition activity. The inhibition assays with CTX-M-14 revealed an IC_50_ value of 13 ± 1.2 µM for Ixazomib, but 67 ± 1.4 µM for Bortezomib (Fig. 3). The fivefold weaker inhibition rate of Bortezomib resembles quite nicely the observation of an elevated degree of disorder for the Pyz-Phe moiety of Bortezomib as discussed above.

### The anion binding site in the vicinity of the substrate binding site

**Figure 4:**
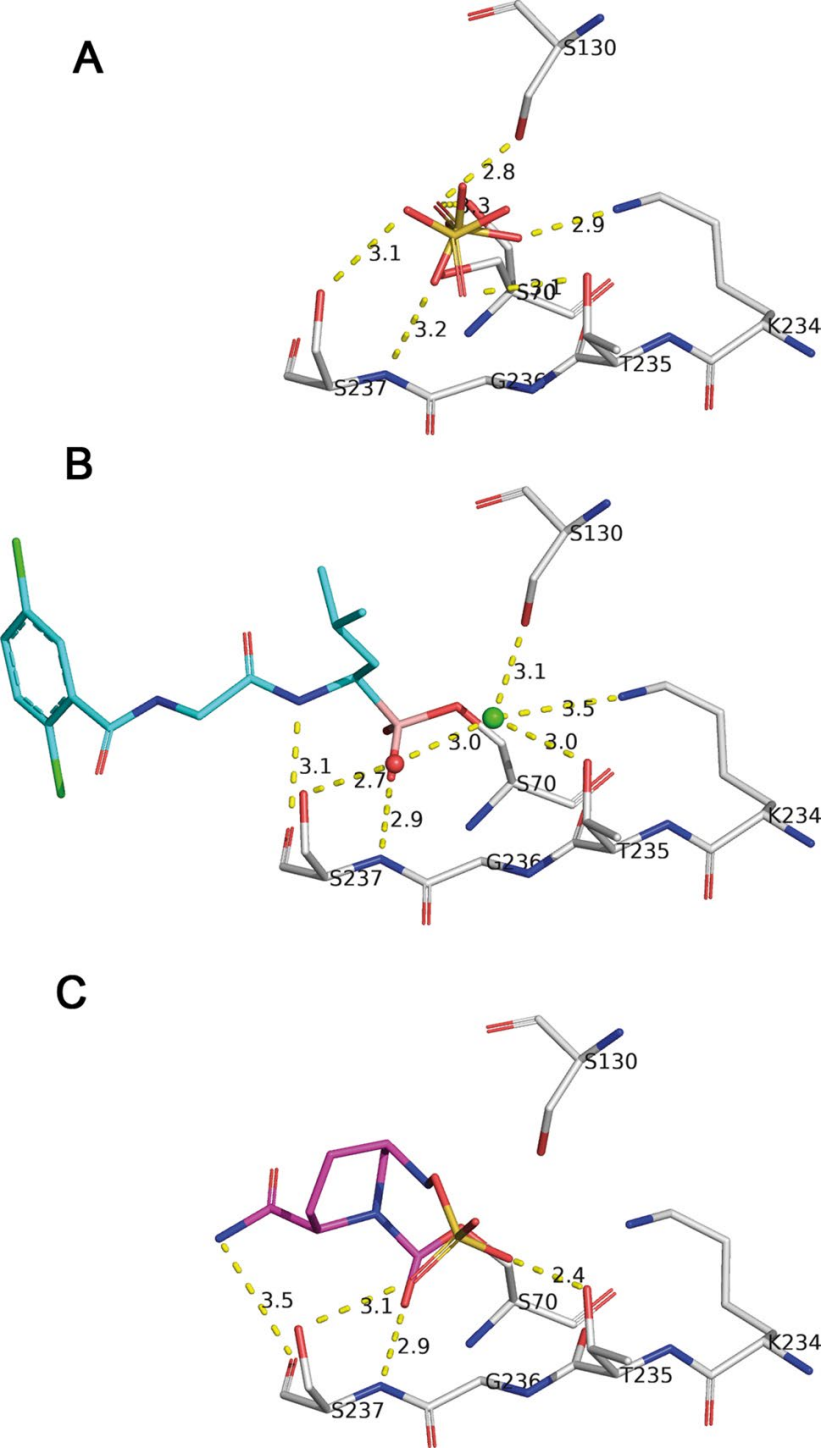
The anion binding site in CTX-M-14 is occupied by a rotational disordered sulphate ion in the native enzyme (**A**). The larger sulphate ion is replaced by a smaller chloride ion upon inhibitor binding and bound Ixazomib is colored in cyan **(B**). This anion binding site is occupied by a sulfonate substituent of DBO (Diazabicyclooctane) inhibitors Avibactam, colored in pink (**C**). The hydrogen bonding networks are represented as yellow dashed lines.

The active site of CTX-M-14 has a crucial anion binding site. In direct vicinity of the Bortezomib/Ixazomib binding site, we identified a chloride ion at the position of the carboxylate of native β-lactam substrates^43^. For the inhibitor-free CTX-M-14 enzyme solved at a resolution of 0.79 Å this position is occupied by a phosphate-ion (PDB code 4UA6). In our crystal structure of the native enzyme at a resolution of 1.0 Å, using phosphate-free crystallization conditions, this site is occupied by a sulphate ion (Fig. 4a). Both anions are too large to bind in presence of the covalently bound Bortezomib/Ixazomib inhibitors, but the smaller chloride ion is still necessary to balance electrostatic requirements of the side chains of Lys73 (5.4 Å) and Lys234 (3.5 Å). The binding pocket for the chloride ion is completed by several polar residues and van der Waals interaction to the leucine moiety (Cβ 3.7–3.8 Å) of the inhibitors (Fig. 4b). Direct hydrogen bonds are donated from the side chains of Ser130 (3.1 Å), Lys234 (3.5 Å) and Thr235 (3.0 Å). Moreover, the chloride ion is indirectly linked to the hydroxyl of Ser237 via a tightly bound structural water molecule.

The direct comparison with the recently approved Diazabicyclooctane (DBO) inhibitors Avibactam and Relebactam revealed that their sulfonate groups occupy the discussed anion binding site (Fig. 4c). The sulfonate was introduced in the course of drug development to increase stability and activity of the DBOs^44^. This characteristic molecular mimicry was also utilized for Vaborbactam and Taniborbactam as the carboxylate appendage of their oxaborine and benzooxaborine moiety, respectively, occupy the same position as well.

### Protonation pattern of Lys73 and Glu166 as key residues for acylation and ester hydrolysis

**Figure 5:**
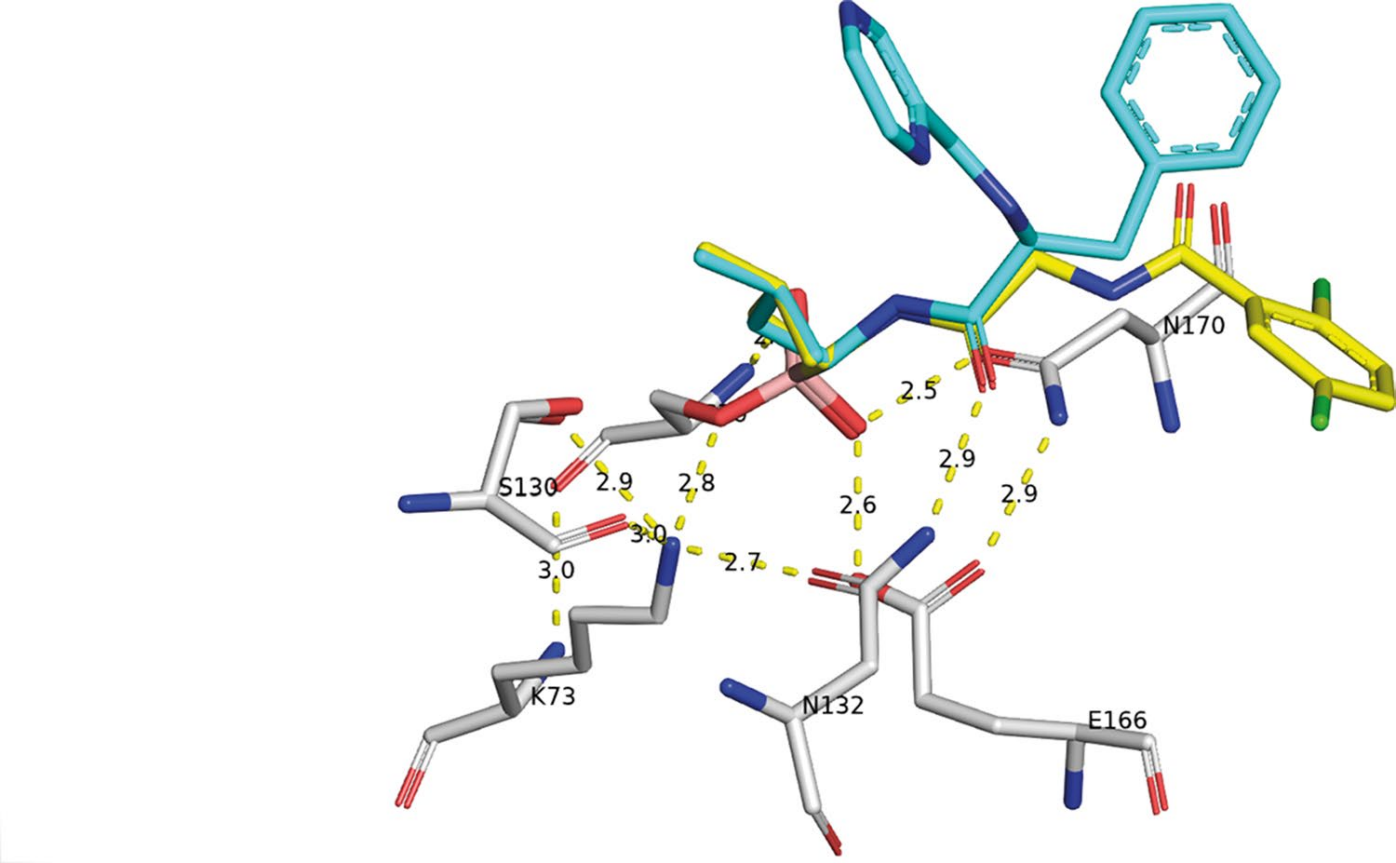
The present hydrogen bonding network in the CTX-M-14 structure complexed with Bortezomib (in cyan) and Ixazomib (in yellow) unravel the protonation state of the active site residues Lysine 73 and Glutamate 166, key residues involved in the acylation mechanism in class A β-lactamases.

Present knowledge of the catalytic cycle is that the side chain of Lys73 (Lys73Nζ) enhances the nucleophilic character of the catalytic Ser70 by hydrogen bond (2.8 Å) to support acyl-enzyme formation. This serine ester is hydrolysed by the catalytic water molecule which is activated by the principal base Glu166. The inhibitor binding mimics the transition state geometry before the acyl enzyme is formed. Ultrahigh-resolution X-ray and neutron diffraction studies, computational simulations and biochemical experiments of enzyme mutants identified the active site hydrogen bonding network spanning from Ser70 to Glu166^41,42^. In the inhibitor-free CTX-M-14 and in the Bortezomib and Ixazomib complexes Lys73Nζ is in the identical position. The hydrogen bonding pattern is completed as hydrogen bond donor to hydroxyl Ser130 (2.9 Å), carbonyl group of Ser130 (3.0 Å), and side chain Oδ1 of Asn132 (2.7 Å). This hydrogen bonding network in the CTX-M-14 structure complexed with Bortezomib and Ixazomib (Fig. 5) nicely resembles the network already shown for a very similar SBL enzyme based on recently presented neutron diffraction data^45^.

The boronate hydroxyl (O2) occupies the position of the deacylating water molecule and is hydrogen bond acceptor to the carboxylic-acid residue Glu166 (2.6 Å). This water molecule and the replacing boronate O2 are hydrogen bond donor to the side chain carboxamide oxygen of Asn170 (2.5 Å). The hydrogen bonding network is further stabilized by a third hydrogen bond donated by the carboxamide nitrogen of Asn170 to the side chain of Glu166 (2.9 Å). The formation of this hydrogen-bond-network is only possible if Glu166 is indeed protonated (neutral) in complex with Bortezomib and Ixazomib. The protonated state of Glu166 has been also observed at ultra-high-resolution for other complexes^42^.

### Bortezomib binding to targeted proteases in comparison to CTX-M-14

**Figure 6:**
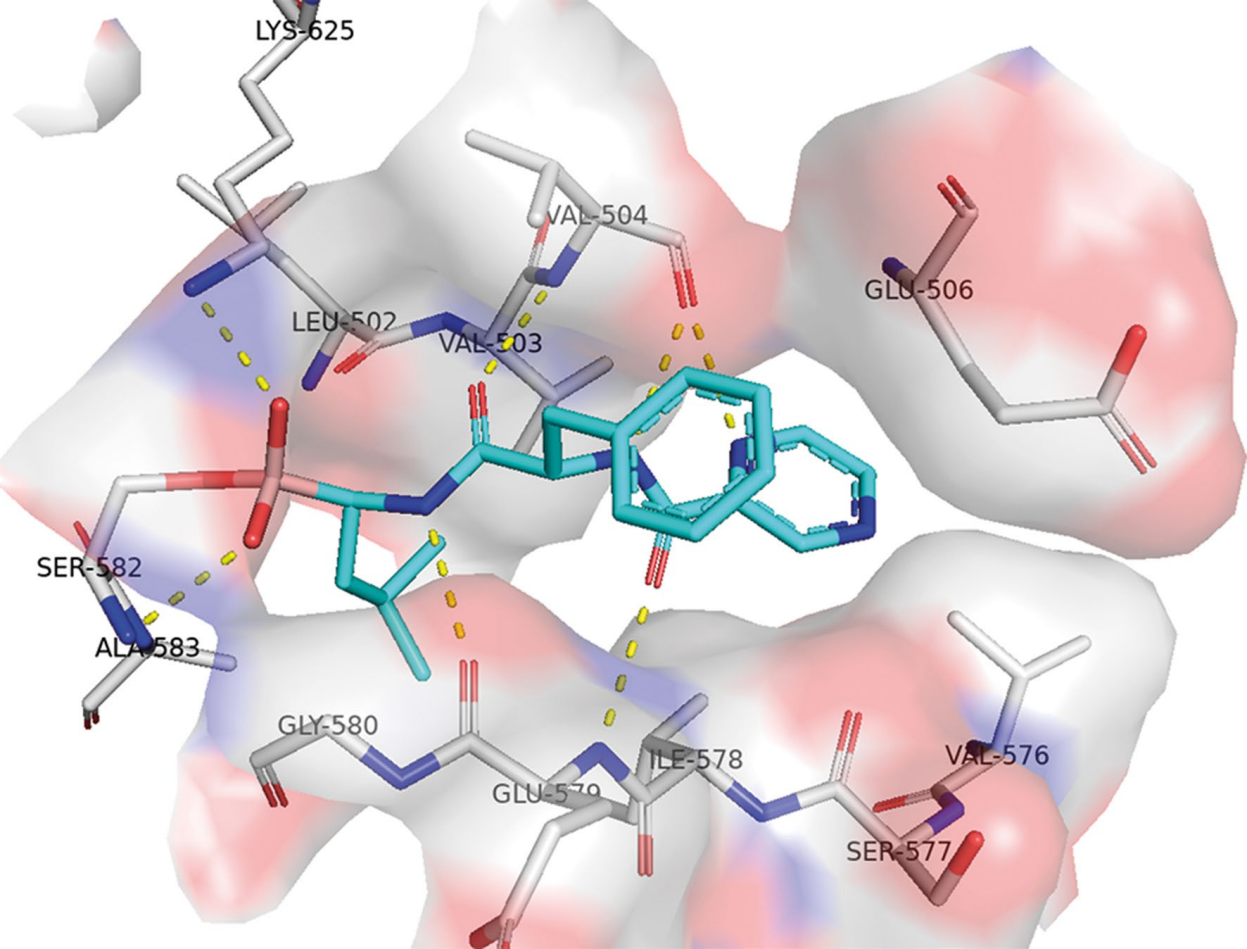
Active site region of the LonC serine protease (PDB entry code 4FWD) with covalently bound Bortezomib to active site serine 582. The pyrazine and leucine moieties are completely buried in the hydrophobic groove in the corresponding S1 and S3 pockets and stabilized by a hydrogen bonding network.

At this point it is reasonable to compare the Bortezomib binding mode for CTX-M-14 and the target Ser/Thr-proteases. In complex with targeted Ser/Thr-proteases^32^ Bortezomib is highly ordered throughout and well defined in the electron density maps even at lower resolution of 1.85 Å^33^. This is consistent with a higher specificity and inhibition capacity towards the targeted proteases in comparison to CTX-M-14. For instance, the complex of Bortezomib with LonC has a 50-fold better IC_50_ value (1.34 µM)^32^ than in complex with CTX-M-14. In the LonC complex, the pyrazine moiety is arrested by two hydrogen bonds and wedges into the S3 side of the pocket (Fig. 6). The corresponding amide group forms two hydrogen bonds, one donated by the main chain nitrogen of Glu579 and a second accepted by the carbonyl oxygen of Val504. The P2 side chain phenylalanine merely points out into the solvent area and is more or less stabilized by van der Waals contacts with Val504 and Glu506. The hydrophobic P1 leucine side chain of Bortezomib protrudes into the S1 side of the pocket. As usual for proteases the P1/P3 residues (leucine and pyrazine) are completely buried in the hydrophobic groove.

### Comparison with approved specific boron-based lactamase inhibitors

It is not surprisingly that acyclic boron-based inhibitors like Bortezomib or Ixazomib and specific cyclic α-acylamino-boronate based β-lactamase inhibitors like Vaborbactam or Taniborbactam share several structural and chemical similarities. All four are basically peptidomimetics imitating a di- or tripeptide core with a central amide-group flanked by the boronic-acid warhead (Fig. 1). The phenylalanine in Bortezomib corresponds to the thiophen/cyclohexyl moiety in Vaborbactam/Taniborbactam, respectively. The boroLeu on the other hand corresponds to oxaborine/benzooxaborine moiety, respectively. The most striking difference is the absence of the pyrazine (Bortezomib) and dichlorBz (Ixazomib) counterpart in Vaborbactam/Taniborbactam. As mentioned above, these moieties are arrested by several hydrogen bonds into the S3 pocket of the targeted proteases and are obviously important for the protease-specificity of Bortezomib and Ixazomib. Variations may provide a tool for specific binding modes and affinities to different Ser/Thr-proteases.

**Figure 7:**
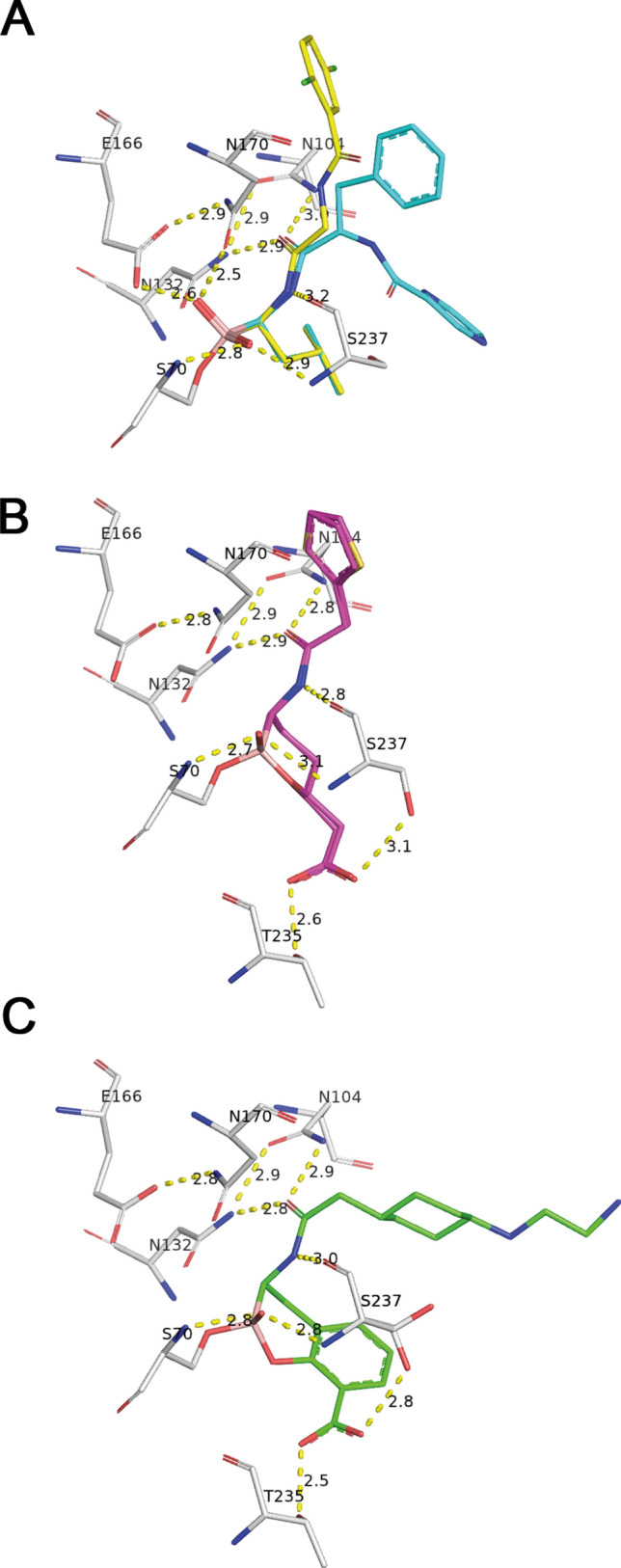
Comparison of the conserved hydrogen bonding network of bound inhibitors and active site residues. Bortezomib and Ixazomib are shown as stick model colored in cyan and yellow, respectively (**A**). The specific α-acylamino-boronate based β-lactamase inhibitor Vaborbactam is colored in pink (**B**) and Taniborbactam in green (**C**).

The structural comparison of the β-lactamase CTX-M-14 complexed with Bortezomib and CTX-M-15 complexed with Vaborbactam/Taniborbactam^25,35^ underlines these similarities as the binding mode is almost identical as well. All involved active-site residues in CTX-M-14/CTX-M-15 are highly conserved and can be superimposed nicely for all three inhibitor complexes. However, it is extremely remarkable that the hydrogen bonding network responsible for the binding (as described above) of all inhibitors coincides very precisely with two fundamental exceptions (Fig. 7). The carboxylate appendage of the oxaborine/benzooxaborine moiety in Vaborbactam/Taniborbactam forms two additional hydrogen bonds with Thr235 and Ser237 side chains. On the other hand, two other strong hydrogen bonds to the carboxylic acid side chain of Glu166 and to the side chain carboxamide oxygen of Asn170 cannot be formed in the Vaborbactam/Taniborbactam complex. In the oxaborine/benzooxaborine moiety the boronate hydroxyl (O2), occupying the position of the deacylating water molecule in the enzyme, was modified by the intramolecular formation of a boronic ester ring. An important task to prevent further hydrolysis and a key for blocking the lactamase is the displacement of the de-acylating water molecule of the enzyme as we see also for Bortezomib and Ixazomib.

The thiophen/cyclohexyl moiety in Vaborbactam/Taniborbactam reveals different orientations. The cyclohexyl moiety of Taniborbactam occupies a similar space as the pyrazine moiety of Bortezomib. The thiophen moiety of Vaborbactam in contrast directs in the position of the phenylalanine part in Bortezomib. For all inhibitors holds true, neither of these parts form direct hydrogen bonds with the enzyme.

A limitation for using Bortezomib or Ixazomib relates to their well-studied inhibition of host serine proteases, thus posing the potential to cause significant treatment emergent adverse events (TEAE) when being used as an anti-infective compound. However, FDA-cleared boronic acid-based β-lactamase inhibitor Vaborbactam only showed a limited number of mild TEAEs during clinical trials^46^, thus showing that off-target effects related to broad serine protease inhibition can be harnessed by specific optimization of compounds for β-lactamase binding.

## Conclusions

We identified and characterized the approved proteasome inhibitors Bortezomib and Ixazomib as serine β-lactamase inhibitors. The inhibition of different classes of peptide hydrolases with boronic-acid based compounds is of general interest for drug discovery approaches. Based on our high-resolution structures it is possible to imply the basis for different binding specificities and resulting differences in their inhibition activity for the CTX-M-14 serine β-lactamase.

The comparative structural analysis of the here presented CTX-M-14 structures in complex with Bortezomib/Ixazomib on the one hand and complexed with their original target proteases on the other hand explains also a higher specificity and inhibition capacity towards the targeted proteases in comparison to CTX-M-14. However, the differences in the binding modes reveal the potential of these boronic-acid based protease inhibitors as promising lead compounds. The determined differences suggest chemical modifications of Bortezomib/Ixazomib to transform them to highly specific β-lactamase inhibitors. This hypothesis is strongly supported by the comparison with already approved and highly specific boronate-based β-lactamase inhibitors like Vaborbactam and Taniborbactam. The comparison reveals that the hydrogen bonding network responsible for the binding of Taniborbactam/Vaborbactam on one side and Bortezomib/Ixazomib on the other side coincides very precisely with only two fundamental additional hydrogen bonds as an exception. The exceptions are obviously responsible for the big differences in higher specificity and inhibition capacity of Taniborbactam/Vaborbactam in comparison to Bortezomib/Ixazomib. Moreover, the anion binding-site in the vicinity of the substrate binding site of CTX-M-14 turned out to be of central significance for the transformation of a weak and unspecific inhibitor towards an inhibitor with high specificity and inhibition capacity. The recently approved and relevant lactamase inhibitors utilize this specific feature.

Further studies applying serial femtosecond crystallography and other biophysical studies are highly required to capture mechanistically relevant transient states, like the pre-covalent Michaelis complex^41,47^. We and others have initiated experiments accordingly^48,49^ and we will continue to track this highly ambitious enterprise.

## Methods

### Molecular cloning, Protein expression and purification

The CTX-M-14 β-lactamase gene was cloned into a pRSET A plasmid and transformed into competent Escherichia coli BL21(DE3) cells (BL21(DE3) pLysS, Novagen, Schwalbach, Germany) as previously described^48^. BL21(DE3) cells were grown in LB medium at 37 °C containing 100 µg/mL ampicillin for plasmid selection. Gene expression was induced by supplementation of IPTG (isopropyl β-D-1-thiogalactopyranoside) to a final concentration of 1 mM at an optical density (OD) of 0.7. Cells were harvested 3 h after induction by centrifugation with 4000 × g at 4 °C. The cell pellet was resuspended in 20 mM MES pH 6 and sonicated for lysis. Cell debris were separated by centrifugation at 17,000 × g for 1 h at 4 °C. Supernatant was supplemented by addition of 1 µl DNase and dialyzed overnight against a large volume of 20 mM MES pH 6 at 4 °C. Dialyzed sample was filtered using a 0.2 µm syringe filter and applied onto a cation exchange column (HiTrap SP XL) using an Äkta Pure chromatography system. The column was pre-equilibrated with 20 mM MES pH 6 and CTX-M-14 was eluted using a gradient over 20 column volumes with 500 mM NaCl, 20 mM MES pH 6. Elution peak was concentrated using a 10 kDa Amicon concentrator to a final CTX-M-14 concentration of 15 mg/mL.

### Protein crystallization and data collection

A freshly prepared protein solution at a concentration of 15 mg/mL was cleared by centrifugation at 12,000 × g. Subsequently, CTX-M-14 crystals appeared within two days employing the sitting-drop vapor-diffusion method at 294 K. 2 µL of protein solution and 2 µL reservoir (26% PEG8000, 200 mM lithium sulphate, 100 mM sodium acetate pH 4.6) were mixed to equilibrate against 100 μL reservoir. Native CTX-M-14 crystals were cryo-protected in mother liquor supplemented with 25% glycerol. To obtain CTX-M-14 complexed with the inhibitors stock solutions of 100 mM Bortezomib in absolute ethanol and Ixazomib in DMSO were prepared. 1 µL of the stock solution was added to droplets with freshly grown crystals. The crystals were soaked for 1 h and afterwards cryo-protected in mother liquor supplemented with 25% glycerol. Subsequently, fished crystals were flash-cooled in liquid nitrogen prior to data collection.

### Diffraction data collection and structure determination

All X-ray diffraction data were collected using synchrotron radiation at 100 K (Beamline P11, Petra III, DESY, Hamburg, Germany)^50^. The diffraction data were processed using the XDS program package^51^ at resolutions of 1.3 Å for the Bortezomib-complex, 1.1 Å for the Ixazomib-complex and 1.0 Å for the inhibitor-free CTX-M-14 enzyme, all in space group P2_1_2_1_2_1_ (Table S1). The structures were determined by molecular replacement with a solvent- and ligand-free structure of CTX-M-14 (PDB entry 6GTH)^48^ as search model using the program phaser from the Phenix software suite (Version 1.19.2.4158; http://www.phenix-online.org/)^52^. Initial refinement was carried out using phenix.refine^53^, with all isotropic atomic displacement parameters (ADPs) set to 20 Å^2^ and using simulated annealing. For the Bortezomib complex subsequently, the ADPs were refined using TLS refinement along with individual isotropic refinement. The structures of the Ixazomib complex and the inhibitor-free enzyme were refined anisotropically for all non-solvent atoms. For manual model re-building the Coot software (Version 0.8.9.2, https://www2.mrc-lmb.cam.ac.uk/personal/pemsley/coot/)^54^ was used and the inhibitor was built into the F_o_—F_c_ difference electron density maps and verified by Polder omit maps^55^. POLYGON^56^ and MolProbity^57^ were used for the validation of the final model. Data collection and structure refinement statistics are listed in Table S1. Figures were generated using ChemDraw (Version 19.0.0.22, PerkinElmer; https://perkinelmerinformatics.com/products/research/chemdraw/ ) and PyMOL^58^ (The PyMOL Molecular Graphics System, Version 2.0 Schrödinger, LLC., https://pymol.org/2/).

### Inhibition Assays

The 50% inhibitory concentration (IC_50_) of CTX-M-14 was determined as the concentration of the inhibitor needed to reduce the initial rate of hydrolysis of the substrate by 50%. The compounds were diluted at concentrations ranging from 750 to 0.00001 µM in PBS buffer at pH 7.4. Diluted enzyme was added to the inhibitor and the solution was incubated at 37 °C for 15 min. After incubation, the substrate Cefotaxime was added at a final concentration of 100 µM and the hydrolysis of the substrate was monitored in 96 well UV-Star Microplates (Greiner Bio-One International GmbH) at 260 nm for 10 min using a Biotek Synergy H1 microplate reader (Biotek Instruments, Inc.). The inhibition assays were performed in triplicates. Initial rates of hydrolysis were plotted against the log of the Bortezomib/Ixazomib concentration and the IC_50_ was calculated by using the nonlinear regression function of GraphPad Prism (Version 5.0.0, GraphPad Software, San Diego, California USA, www.graphpad.com).

## Supplementary Information

Supplementary Information.

## Abbreviations

**CTX-M-14**: Cefotaximase (CTX) firstly isolated in Munich (M), Variant 14
**BL**: β-lactamase
**ESBL**: Extended-spectrum β-lactamase
**SBL**: Serine-reactive β-lactamase
**DBO**: Diazabicyclooctane
