# Supplementary material for: Structural basis to repurpose boron-based proteasome inhibitors Bortezomib and Ixazomib as β-lactamase inhibitors
Source: Sci Rep. 2022 Apr 1;12:5510. doi: 10.1038/s41598-022-09392-6 (PMC8976068; doi:10.1038/s41598-022-09392-6)
Supplement: Supplementary file 1 — Supplementary Information. [file 41598_2022_9392_MOESM1_ESM.pdf]

Supplement

Table S1: Data collection and refinement statistics

	CTX-M14 Bortezomib	CTX-M14 Ixazomib	CTX-M14
**Data collection**			
X-ray source	P11, Petra III, DESY	P11, Petra III, DESY	P11, Petra III, DESY
Detector	Pilatus 6M	Pilatus 6M	Pilatus 6M
Space group	$P2_12_12_1$	$P2_12_12_1$	$P2_12_12_1$
Cell dimensions			
a, *b*, *c* (Å)	41.3, 62.4, 86.3	41.5 62.4 87.4	41.4 62.5 86.4
Wavelength (Å)	1.0330	1.0330	0.9762
Resolution (Å)	35.46-1.30 (1.35-1.30)	37.46-1.14 (1.18-1.14)	29.88-1.00 (1.04-1.00)
Total reflections	390744 (38038)	1035046 (93282)	1461592 (121485)
Total unique reflections	55064 (5308)	83198 (8214)	121023 (11775)
Redundancy	7.1 (7.2)	12.4 (11.4)	12.1 (10.3)
Wilson *B*-factor ($Å^2$)	9.84	8.08	7.02
R_{meas}	0.073 (0.527)	0.052 (0.263)	0.038 (0.247)
$CC_{1/2}$	1 (0.971)	1 (0.982)	1 (0.982)
$I/\sigma I$	16.89 (3.59)	29.01 (8.06)	36.49 (7.83)
Completeness (%)	99.0 (93.0)	99.98 (99.96)	99.63 (97.83)
**Refinement**			
Reflections used	55062 (5308)	83198 (8214)	121018 (11774)
Reflection used for R_{free}	1993 (192)	2100 (208)	6051 (589)
R_{work}	0.1278 (0.2131)	0.1316 (0.1328)	0.1103 (0.1070)
R_{free}	0.1439 (0.2356)	0.1430 (0.1623)	0.1222 (0.1329)
No. atoms	2502	2515	2608
Protein	1995	2053	2070
Ligand/ion	75	113	71
Water	468	392	490
Average *B*-factor ($Å^2$)	15.20	11.00	11.15
Macromolecules	11.70	9.14	8.45
Ligands	28.61	15.49	17.01
Water	29.01	21.04	22.08
R.m.s deviations			
Bond lengths (Å)	0.010	0.007	0.019
Bond angles (º)	1.10	1.09	1.62
Ramachandran			
favored (%)	98.47	98.08	98.46
allowed (%)	1.15	1.54	1.15
outliers (%)	0.38	0.38	0.38
PDB code	7Q0Y	7Q11	7Q0Z

Statistics for the highest-resolution shell are shown in parentheses.
